# Obesity as a risk factor for COVID‐19 mortality in women and men in the UK biobank: Comparisons with influenza/pneumonia and coronary heart disease

**DOI:** 10.1111/dom.14199

**Published:** 2020-10-11

**Authors:** Sanne A. E. Peters, Stephen MacMahon, Mark Woodward

**Affiliations:** ^1^ George Institute for Global Health Imperial College London London UK; ^2^ Julius Center for Health Sciences and Primary Care, University Medical Center Utrecht Utrecht University Utrecht The Netherlands; ^3^ George Institute for Global Health University of New South Wales Sydney New South Wales Australia; ^4^ Department of Epidemiology Johns Hopkins University Baltimore Maryland USA

**Keywords:** body composition, cardiovascular disease, cohort study

## Abstract

Obesity is associated with severe COVID‐19 outcomes, yet, it is unclear whether the risk of COVID‐19 mortality associated with obesity is similar between the sexes. We used data from the UK Biobank to assess the risk of COVID‐19 mortality associated with various anthropometric measures in women and men. To put these results in context, we also compared these estimates with those for mortality from influenza/pneumonia and coronary heart disease (CHD). The analyses included 502 493 individuals (54% women), of whom 410 (36% women) died from COVID‐19, 549 (36% women) died from influenza/pneumonia and 3355 (19% women) died from CHD. A higher body mass index (BMI), waist circumference, waist‐to‐hip ratio and waist‐to‐height ratio were each associated with a greater risk of death from COVID‐19, influenza/pneumonia and CHD in both sexes, with the exception of the association between higher BMI and the risk of influenza/pneumonia death in men. A higher BMI was associated with a stronger risk of COVID‐19 mortality in women than men; the women‐to‐men ratio of hazard ratios was 1.20 (95% confidence interval 1.00; 1.43). This study demonstrates the role of obesity in COVID‐19 mortality and shows that the relative effects of a higher BMI on COVID‐19 mortality may be stronger in women than men.

## 1 INTRODUCTION

Although women and men are approximately as likely to be infected by coronavirus disease 2019 (COVID‐19), men have higher death rates from COVID‐19 in almost all countries where data are available.^1^ Obesity has been identified as one of the key factors associated with severe COVID‐19 outcomes,^2^, ^3^ some of which may be explained by the adverse effects of obesity on diabetes. However, it is unclear whether the risk of COVID‐19 mortality associated with obesity is similar between the sexes. We thus assessed the risk of COVID‐19 mortality associated with various anthropometric measures in women and men in the UK Biobank. For comparability, we also evaluated the association of these measures with mortality from influenza or pneumonia, the leading causes of death from respiratory disease in usual circumstances, and coronary heart disease (CHD), a condition which has a well‐established association with obesity.

## 2 METHODS

The UK Biobank is a prospective, population‐based cohort study of women and men aged 40 to 69 years at baseline between 2006 and 2010.^4^ Follow‐up for cause‐specific mortality was conducted to June 30, 2020 through linkage with the National Death Register.

Cox regression was used to estimate the sex‐specific hazard ratios (HRs) and 95% confidence intervals (CIs) for mortality from COVID‐19, influenza/pneumonia and CHD for overweight and obesity (defined as a body mass index [BMI] ≥25 to <30 kg/m^2^ and ≥30 kg/m^2^, respectively) and an overall 1‐standard deviation (SD) increase in BMI (SD 4.8), waist circumference (SD 13.5), waist‐to‐hip ratio (SD 0.09) and waist‐to‐height ratio (SD 0.08). Influenza and pneumonia were taken together to avoid unreliable estimation due to small numbers. CIs were estimated using floating absolute risks.^5^ Adjustments were made for age, smoking status (never/ex/current), socio‐economic status (determined using the Townsend index of area deprivation) and ethnicity (white or not). Interactions between each variable and sex were added to the model, so as to obtain the women‐to‐men ratio of HRs (RHRs) for each risk factor.^6^ Penalized smoothing splines were used to examine the shape of associations between BMI and the study endpoints. In secondary analyses, we additionally adjusted for diabetes. We also stratified our analyses on COVID‐19 mortality for ethnicity because a previous UK Biobank analysis suggested that the association between BMI and the risk of testing positive for COVID‐19 may vary by ethnicity.^7^ Analyses used R version 3.3.0.

### 2.1 Ethics

This research was conducted using the UK Biobank Resource (application No 2495). Permission to use the UK Biobank Resource was approved by the access subcommittee of the UK Biobank Board. UK Biobank has obtained Research Tissue Bank approval from its governing research ethics committee, as recommended by the National Research Ethics Service. No separate ethical approval was required. The study was conducted in accordance with the principles of the Declaration of Helsinki.

## 3 RESULTS

Of the 502 493 individuals (54% women) included in the analyses, 410 (36% women) died from COVID‐19, 549 (36% women) died from influenza/pneumonia, and 3355 (19% women) died from CHD during a median follow‐up of 11.2 years.

**FIGURE 1:**
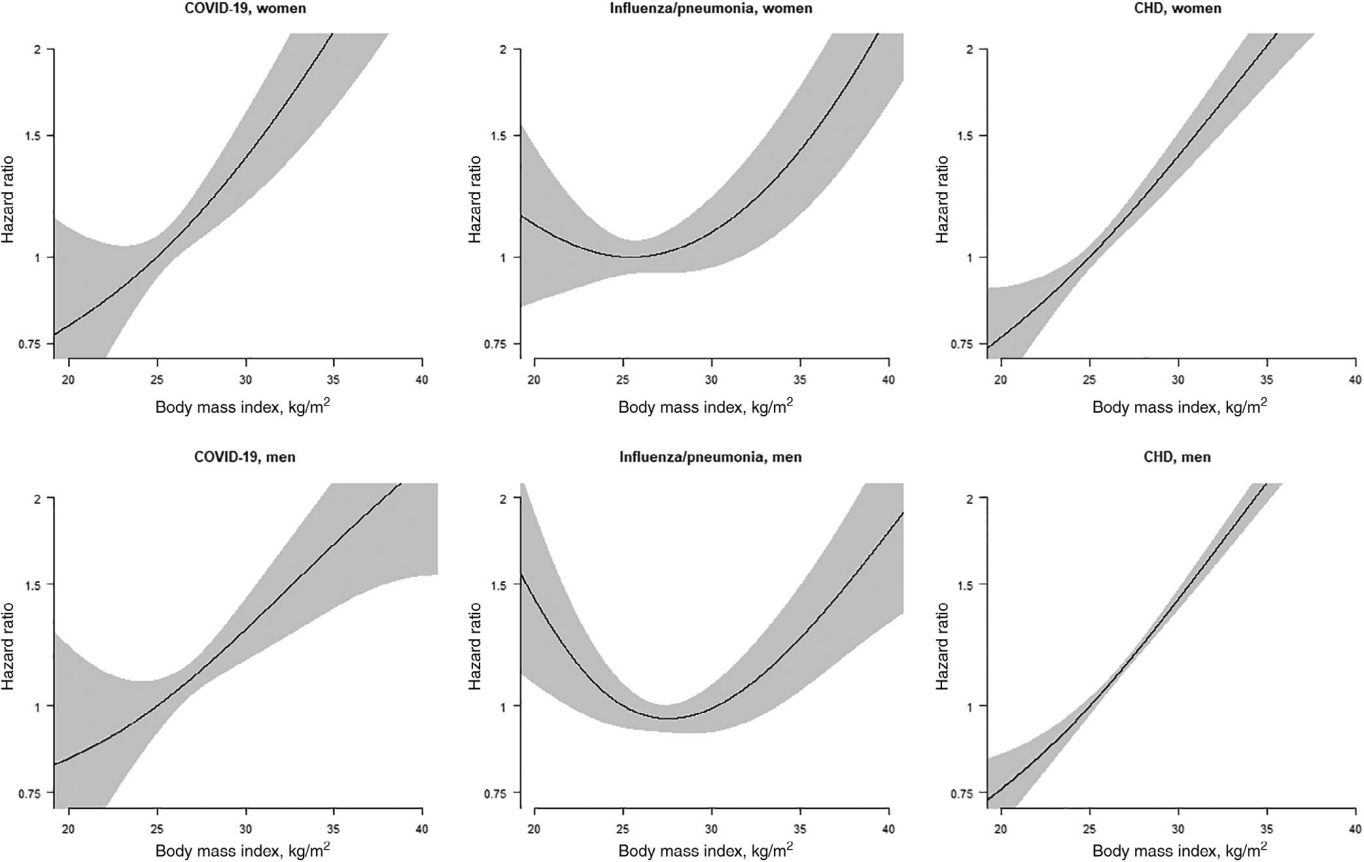
Penalized spline plots with adjusted hazard ratios and 95% confidence intervals for death from COVID‐19, influenza/pneumonia or coronary heart disease (CHD) associated with body mass index. Analyses are adjusted for age, Townsend index, smoking status and ethnicity

A higher BMI, waist circumference, waist‐to‐hip ratio and waist‐to‐height ratio were each associated with a greater risk of death from COVID‐19, influenza/pneumonia and CHD in both sexes (Table 1). The only exception was the association between higher BMI and the risk of influenza/pneumonia death in men, which may be attributable to the apparent underlying U‐shaped relationship (Figure 1). The shape and magnitude of the relationship between BMI and COVID‐19 mortality was similar to the relationship with CHD mortality. Obesity was associated with an increased risk of each cause of death in both sexes, except for influenza/pneumonia in men.

**TABLE 1:** Adjusted hazard ratios (HRs) and women‐to‐men ratios of HRs with 95% confidence intervals for death from COVID‐19, influenza/pneumonia or coronary heart disease associated with body mass index, waist circumference, waist‐to‐hip ratio and waist‐to‐height ratio

	COVID‐19			Influenza/pneumonia			Coronary heart disease		
	Women	Men	Ratio of HRs	Women	Men	Ratio of HRs	Women	Men	Ratio of HRs
**Adiposity**									
BMI	1.51 (1.34; 1.71)	1.26 (1.11; 1.44)	1.20 (1.00; 1.43)	1.31 (1.17; 1.47)	1.11 (0.98; 1.24)	1.19 (1.01; 1.40)	1.39 (1.31; 1.48)	1.43 (1.38; 1.49)	0.97 (0.91; 1.05)
Waist circumference	1.66 (1.41; 1.94)	1.36 (1.18; 1.57)	1.22 (0.98; 1.51)	1.41 (1.22; 1.62)	1.19 (1.05; 1.34)	1.18 (0.98; 1.42)	1.60 (1.48; 1.72)	1.52 (1.46; 1.58)	1.05 (0.96; 1.14)
Waist‐to‐hip ratio	1.34 (1.23; 1.47)	1.57 (1.37; 1.79)	0.86 (0.73; 1.01)	1.37 (1.25; 1.50)	1.24 (1.07; 1.43)	1.11 (0.94; 1.32)	1.41 (1.36; 1.46)	1.58 (1.52; 1.65)	0.89 (0.84; 0.94)
Waist‐to‐height ratio	1.60 (1.39; 1.84)	1.35 (1.18; 1.55)	1.18 (0.97; 1.43)	1.43 (1.26; 1.61)	1.30 (1.16; 1.46)	1.09 (0.93; 1.29)	1.58 (1.48; 1.68)	1.55 (1.49; 1.61)	1.02 (0.94; 1.10)
**BMI categories**									
Healthy weight	1.00 (0.71; 1.40)	1.00 (0.74; 1.36)	1.00 (0.63; 1.58)	1.00 (0.79; 1.27)	1.00 (0.81; 1.24)	1.00 (0.73; 1.38)	1.00 (0.86; 1.17)	1.00 (0.91; 1.10)	1.00 (0.83; 1.20)
Overweight	1.25 (0.94; 1.65)	1.31 (1.10; 1.57)	0.95 (0.68; 1.33)	0.72 (0.55; 0.94)	0.79 (0.67; 0.93)	0.91 (0.67; 1.25)	1.23 (1.08; 1.41)	1.25 (1.18; 1.33)	0.98 (0.85; 1.14)
Obese	2.21 (1.69; 2.88)	1.78 (1.44; 2.21)	1.24 (0.88; 1.74)	1.48 (1.17; 1.88)	1.16 (0.96; 1.40)	1.28 (0.95; 1.74)	2.19 (1.93; 2.49)	2.21 (2.08; 2.35)	0.99 (0.86; 1.14)

Abbreviation: BMI, body mass index. Analyses are adjusted for age, Townsend index, smoking status, and ethnicity. HRs for continuous variables are per 1‐SD higher value, taking the overall SD from the sex‐combined baseline data. HRs for BMI categories are presented on a floating absolute scale, with the healthy weight group as the reference group.

A 1‐SD higher BMI was associated with a stronger risk of COVID‐19 and influenza/pneumonia mortality in women than men: HRs for COVID‐19 mortality were 1.51 (95% CI 1.34; 1.71) in women and 1.26 (95% CI 1.11; 1.44) in men, leading to a women:men RHR of 1.20 (95% CI 1.00; 1.43). Similar results were seen for waist circumference and waist‐to‐height ratio, although the RHRs were both marginally non‐significant. The association of higher values of waist‐to‐hip ratio with COVID‐19 mortality was greater for men than women (HRs of 1.34 [95% CI 1.23; 1.47] in women and 1.57 [95% CI 1.37; 1.79] in men), with an RHR of 0.86 (95% CI 0.73; 1.01).

Virtually the same sex difference relating to a 1‐SD higher BMI, to that for COVID‐19, was found for influenza/pneumonia: the HR was 1.19 (95% CI 1.01; 1.40). In this case, the three other measures of adiposity showed similar sex differences, although they did not reach traditional levels of statistical significance. However, for CHD, there was no evidence of a sex difference in the effects of any measure of adiposity. The magnitude of sex differences in the effect of obesity, compared to healthy weight, was roughly the same as for higher values of BMI, for all three causes of death. Results were broadly similar after adjustment for diabetes (Table S1).

Compared with their white counterparts, the association between obesity measures and COVID‐19 mortality was stronger among non‐white women, but broadly similar in non‐white men (Table S2). The risk of COVID‐19 mortality associated with obesity was 8.55 (95% CI 4.02; 18.19) in non‐white women and 1.51 (95% CI 0.68; 3.36) in non‐white men; the corresponding RHR was 5.67 (95% CI 1.89; 17.06).

## 4 DISCUSSION

This population‐based study of over 500 000 women and men in the UK Biobank shows that higher levels of adiposity measures were associated with a higher risk of death from COVID‐19, of an order of magnitude similar to that seen for other respiratory diseases and CHD. The findings for COVID‐19 are in agreement with previous studies that highlighted the role of obesity in COVID‐19 severity and mortality, although these studies did not investigate the sex‐specific associations.^2^, ^3^ They also add to the findings of previous studies in the UK Biobank that showed that both BMI and waist circumference were associated with an increased chance of testing positive for COVID‐19, especially among non‐white people.^7^ We show that the associations between higher BMI, and obesity compared to healthy weight, with death from COVID‐19 and death from influenza or pneumonia, were each approximately 20% greater in women than men. Our analyses also suggest that the risk of COVID‐19 mortality associated with obesity may be particularly strong among women from non‐white backgrounds, although the number of non‐white individuals was relatively small. In agreement with our previous meta‐analysis,^8^ there was no sex difference in the effects of adiposity on death from CHD. By and large, results were similar for the other three measures of adiposity considered. We are unable to explain the exception to this general agreement in that we found a higher effect on COVID‐19 mortality of waist‐to‐hip ratio in men than women; this requires further study. Otherwise, given that the association between BMI and COVID‐19 mortality was stronger, not weaker, in women than men, the greater risk of death from COVID‐19 in men is unlikely to be attributable to a greater relative impact of obesity on death from COVID‐19 in men. Although our analyses relied on single measurements of obesity markers at study baseline, we anticipate that the impact of any changes in obesity levels during follow‐up and the risk of COVID‐19 would be similar for women and men.

These results indicate that, if causal, obesity prevention strategies aimed at reducing the burden of several chronic diseases should also lead to better outcomes among both women and men affected by COVID‐19. Furthermore, the striking similarity in most of the observed effects of adiposity on COVID‐19 and the other two respiratory diseases considered, both in sex‐specific and sex‐comparative terms, suggests that obesity is likely to be a key driver of mortality in any future viral epidemic, particularly amongst women.

## CONFLICTS OF INTEREST

None declared.

## AUTHOR CONTRIBUTIONS

All authors were involved in the design of the study. S.P. carried out the statistical analyses and wrote the first draft of the paper, with support from S.M. and M.W. All authors contributed to further drafts and approved the final manuscript. The corresponding author attests that all listed authors met the authorship criteria and that no others meeting the criteria have been omitted. S.P. is the guarantor.

5

### PEER REVIEW

The peer review history for this article is available at https://publons.com/publon/10.1111/dom.14199.

## Supporting information


**Table S1** Adjusted hazard ratios (HRs) and women‐to‐men ratios of HRs with 95% confidence intervals for death from COVID‐19, influenza/pneumonia, or coronary heart disease associated with body mass index, waist circumference, waist‐to‐hip ratio and waist‐to‐height ratio
**Table S2** Adjusted hazard ratios (HRs) and women‐to‐men ratios of HRs with 95% confidence intervals for death from COVID‐19 associated with body mass index, waist circumference, waist‐to‐hip ratio and waist‐to‐height ratio, stratified by ethnicity.

## Data Availability

Researchers can apply to use the UK Biobank resource and access the data used. No additional data are available.
