# Supplementary material for: Obesity as a risk factor for COVID‐19 mortality in women and men in the UK biobank: Comparisons with influenza/pneumonia and coronary heart disease
Source: Diabetes Obes Metab. 2020 Oct 11;23(1):258–62. doi: 10.1111/dom.14199 (PMC7536945; doi:10.1111/dom.14199)
Supplement: Supplementary file 1 — Table S1 Adjusted hazard ratios (HRs) and women‐to‐men ratios of HRs with 95% confidence intervals for death from COVID‐19, influenza/pneumonia, or coronary heart disease associated with body mass index, waist circumference, waist‐to‐hip ratio and waist‐to‐height ratio Table S2 Adjusted hazard ratios (HRs) and women‐to‐men ratios of HRs with 95% confidence intervals for death from COVID‐19 associated with body mass index, waist circumference, waist‐to‐hip ratio and waist‐to‐height ratio, stratified by ethnicity. [file DOM-23-258-s001.docx]

**Supplementary Appendix**

**eTable 1 Adjusted hazard ratios (HRs) and women-to-men ratios of HRs with 95% confidence intervals for death from COVID-19, influenza/pneumonia, or coronary heart disease associated with body mass index, waist circumference, waist-to-hip ratio, and waist-to-height ratio**

|  | **COVID-19** | | | **Influenza/pneumonia** | | | **Coronary heart disease** | | |
| --- | --- | --- | --- | --- | --- | --- | --- | --- | --- |
|  | **Women** | **Men** | **Ratio of HRs** | **Women** | **Men** | **Ratio of HRs** | **Women** | **Men** | **Ratio of HRs** |
| **Adiposity** |  |  |  |  |  |  |  |  |  |
| *BMI* | 1.47 (1.29; 1.67) | 1.21 (1.06; 1.38) | 1.22 (1.01; 1.47) | 1.19 (1.06; 1.35) | 1.00 (0.89; 1.13) | 1.19 (1.01; 1.42) | 1.22 (1.14; 1.30) | 1.29 (1.24; 1.34) | 0.95 (0.88; 1.02) |
| *Waist circumference* | 1.61 (1.36; 1.90) | 1.30 (1.12; 1.50) | 1.24 (0.99; 1.54) | 1.26 (1.09; 1.46) | 1.07 (0.95; 1.22) | 1.17 (0.97; 1.42) | 1.35 (1.25; 1.47) | 1.35 (1.30; 1.41) | 1.00 (0.92; 1.09) |
| *Waist-to-hip ratio* | 1.33 (1.20; 1.46) | 1.52 (1.31; 1.75) | 0.87 (0.73; 1.04) | 1.31 (1.17; 1.48) | 1.12 (0.96; 1.30) | 1.18 (0.97; 1.42) | 1.36 (1.29; 1.43) | 1.44 (1.38; 1.52) | 0.94 (0.87; 1.01) |
| *Waist-to-height ratio* | 1.56 (1.35; 1.80) | 1.29 (1.12; 1.49) | 1.21 (0.99; 1.48) | 1.30 (1.14; 1.48) | 1.18 (1.05; 1.33) | 1.10 (0.92; 1.31) | 1.37 (1.28; 1.47) | 1.39 (1.33; 1.45) | 0.99 (0.91; 1.07) |
| **BMI categories** |  |  |  |  |  |  |  |  |  |
| *Healthy weight* | 1.00 (0.71; 1.41) | 1.00 (0.74; 1.36) | 1.00 (0.63; 1.58) | 1.00 (0.78; 1.28) | 1.00 (0.81; 1.24) | 1.00 (0.72; 1.39) | 1.00 (0.85; 1.17) | 1.00 (0.91; 1.10) | 1.00 (0.83; 1.20) |
| *Overweight* | 1.22 (0.92; 1.61) | 1.29 (1.08; 1.54) | 0.95 (0.68; 1.32) | 0.68 (0.52; 0.88) | 0.75 (0.64; 0.89) | 0.90 (0.66; 1.23) | 1.13 (1.00; 1.29) | 1.17 (1.11; 1.24) | 0.97 (0.84; 1.12) |
| *Obese* | 2.02 (1.53; 2.67) | 1.64 (1.31; 2.04) | 1.23 (0.87; 1.76) | 1.18 (0.91; 1.51) | 0.95 (0.78; 1.16) | 1.24 (0.90; 1.70) | 1.60 (1.39; 1.83) | 1.76 (1.65; 1.88) | 0.91 (0.78; 1.05) |

Analyses are adjusted for age, Townsend index, smoking status, ethnicity, and diabetes. HRs for continuous variables are per 1-SD higher value, taking the overall SD from the sex-combined baseline data. HRs for BMI categories are presented on a floating absolute scale, with the healthy weight group as the reference group.

**eTable 2 Adjusted hazard ratios (HRs) and women-to-men ratios of HRs with 95% confidence intervals for death from COVID-19 associated with body mass index, waist circumference, waist-to-hip ratio, and waist-to-height ratio, stratified by ethnicity**

|  | **White** | | | **Non-White** | | |
| --- | --- | --- | --- | --- | --- | --- |
|  | **Women** | **Men** | **Ratio of HRs** | **Women** | **Men** | **Ratio of HRs** |
| **BMI** | 1.46 (1.28; 1.66) | 1.28 (1.12; 1.46) | 1.14 (0.94; 1.38) | 1.90 (1.36; 2.65) | 1.26 (0.79; 2.00) | 1.51 (0.85; 2.66) |
| **Waist circumference** | 1.59 (1.35; 1.88) | 1.37 (1.18; 1.58) | 1.16 (0.93; 1.45) | 2.30 (1.38; 3.82) | 1.44 (0.89; 2.33) | 1.60 (0.79; 3.23) |
| **Waist-to-hip ratio** | 1.33 (1.21; 1.46) | 1.57 (1.37; 1.80) | 0.85 (0.72; 1.00) | 1.83 (0.94; 3.56) | 1.65 (0.93; 2.93) | 1.11 (0.46; 2.68) |
| **Waist-to-height ratio** | 1.52 (1.31; 1.76) | 1.37 (1.19; 1.58) | 1.11 (0.90; 1.36) | 2.39 (1.58; 3.62) | 1.34 (0.83; 2.14) | 1.79 (0.95; 3.35) |
| **BMI categories** |  |  |  |  |  |  |
| **Healthy weight** | 1.00 (0.71; 1.41) | 1.00 (0.73; 1.37) | 1.00 (0.63; 1.60) | 1.00 (0.14; 7.14) | 1.00 (0.38; 2.67) | 1.00 (0.11; 9.00) |
| **Overweight** | 1.16 (0.86; 1.56) | 1.34 (1.10; 1.61) | 0.87 (0.61; 1.23) | 3.50 (1.32; 9.30) | 1.42 (0.78; 2.56) | 2.47 (0.79; 7.75) |
| **Obese** | 1.97 (1.48; 2.61) | 1.87 (1.50; 2.34) | 1.05 (0.73; 1.50) | 8.55 (4.02; 18.19) | 1.51 (0.68; 3.36) | 5.67 (1.89; 17.06) |

Analyses are adjusted for age, Townsend index, and smoking status. HRs for continuous variables are per 1-SD higher value, taking the overall SD from the sex-combined baseline data. HRs for BMI categories are presented on a floating absolute scale, with the healthy weight group as the reference group.
